# Pennsylvania State Veterinary Medical Association

**Published:** 1896-05

**Authors:** W. G. Benner

**Affiliations:** Recording Secretary


Secretary.

### PENNSYLVANIA STATE VETERINARY MEDICAL ASSOCIATION.

(Concluded from page 345.)

*Afternoon session* called to order by the President at 2.45 P.M, on March 3.

Dr. Hoskins called up the amendment to Art. V., Sec. 2, of the By-laws. Discussed by Dr. Ridge, who favored adhering to old rule. Dr. Hoskins thought we had given the members ample and fair trial; that inasmuch as the members do not respond to Secretary's letter, but sharing the profits, he wanted the amendment enforced. Dr. Zuill considered the action taken at the meeting illegal on account of absence of quorum; but the chair decided that the meeting was regularly called, therefore the action was legal. Dr. Zuill appealed from the decision, but the Association upheld the chair by a rising vote. Dr. Stanton favored something more stimulating in a literary way to the delinquents. Dr. Goentner considered it unfair to expel a member on account of absence at three consecutive meetings as long as dues remained paid, as some might be unable to attend on account of inability to secure a substitute. Dr. Rechtenwald said if the amendment was enforced he would be compelled to resign. Dr. Hoskins thought it would be an incentive to better attendance. Dr. Harger thought if a member had enough interest in the welfare of the Association he could not be kept away, barring sickness; but coercion to attendance was a bad incentive. The adoption of such an amendment would be equivalent to punishment for a personal transgression. Dr. Zuill thought it unconstitutional; that if a man pays his dues he is a member and could not be compelled to attend. The question was finally decided by a rising vote, 12 for and 16 against. Dr. Kooker then moved that Section 2, Article V., be stricken from the By-laws, which was carried as a resolution by Drs. Kooker, Zuill, and Harger.

Dr. Hoskins then, upon motion, read the report of the Committee on Legislation, which was accepted by the Association and ordered placed on file, and the Committee discharged. Discussion followed by Drs. Zuill, Hoskins, and Rechtenwald upon the matter of false registration, examination of applicants by Board of Examiners, insufficient remuneration, etc. It was moved and seconded that the Association would give the Committee on Legislation proper support in conducting necessary litigation.

The report of Committee on Sanitary Science and Police, on account of absence of chairman, Dr. Adams, was postponed until next day.

General discussion on subject of "Tuberculosis of Cattle" was then in order, and was opened by the chairman, Dr. Pearson. He spoke on the work of the State Live-stock Sanitary Commission, the plan adopted, etc. He believed in dealing with it conservatively as there is considerable opposition. Further discussed by Drs. Bridge, Conard, Jobson, Eves. Dr. Pearson, upon inquiry, answered that the sanitary measures adopted to prevent the extension of the disease, after once testing, were thorough cleansing with hot water and soap, afterward spraying with solution of bichloride of mercury (1 : 1000), sunlight, and ventilation. Dr. Zuill again claimed that tuberculin is not an infallible agent, as it would give reactions in diseases other than tuberculosis. Dr. Hoskins said that the dairymen ought to be more careful as to personal cleanliness. Dr. Miller believed in thorough cleanliness, and described the sanitary arrangements at Fairfield Dairy, Essex County, N. J. Dr. Eves, of Delaware, believed in dairy-inspection as well as milk-inspection, where the former is not admissible.

The meeting then adjourned at 5.10 P.M., and closed the first day's session.

*Second day's session.* Called to order at 10.15 A.M., March 4, Vice-President Ridge in the chair.

Committee on Sanitary Science and Police, Dr. Conard, chairman, reported that outbreaks of glanders, tuberculosis, hog-cholera, the usual influenza, strangles, and epizoötic abortion had occurred; the latter he considered the cause of more loss to dairymen than tuberculosis. Upon motion, the report of the Committee was received and ordered placed on file, and the Committee discharged. Discussion opened by Dr. Hoskins, followed by Drs. Goentner, Conard, Harger, Zuill, Rechtenwald, Michener, Jobson.

Report of Committee on Legislation, by Dr. Hoskins, then followed. Report was accepted, on motion, and ordered filed, and Committee discharged. It was moved by Dr. Kooker and seconded by Dr. T. B. Rayner, that a vote of thanks be tendered Dr. Hoskins for his valuable services in the interest of the profession, which was carried unanimously by a rising vote; Dr. Hoskins accepted the offering gratefully.

The reading of papers being in order, the President called upon Dr. J. C. Michener for his paper on the "Pleasure of Being a Veterinarian;" Dr. James B. Rayner on "Osteoporosis;" Dr. W. L. Rhoads on "Pathogenesis and Development of Disease;" Dr. W. L. Zuill, "Description of the Operation of Cunean Tenotomy;" Dr. Harger, "A Description of the Operation of Median Neurotomy;" Dr. Pearson, "Description on the Use of Barium Chloride." The papers as well as the descriptions, to say the least, were exceedingly edifying and enjoyed by all present. The discussion that followed on the several topics was participated in by Drs. Kooker, Hoskins, Michener, Lusson, Conard, Williams, Goentner, Miller, Ridge, Rechtenwald, Helmer, Harger, Eves, and Zuill.

Dr. C. H. Magill handed in his resignation, which was, in accordance with the rules, received and referred to the Board of Trustees.

Dr. W. B. Atkinson, editor and publisher of *Public Health*, asked for the proceedings of the meetings for publication, which, upon motion, were ordered to be furnished.

W. G. BENNER, V.S.,
Recording Secretary.

